# Structural Characterization and Magnetic Behavior Due to the Cationic Substitution of Lanthanides on Ferrite Nanoparticles

**DOI:** 10.3390/nano14110971

**Published:** 2024-06-03

**Authors:** Cristóbal Pinto García, Arianne Maine, Rodrigo A. Valenzuela-Fernández, Álvaro Aliaga Cerón, Patricia Barahona Huenchumil, Octavio Peña, Inmaculada Álvarez-Serrano, Andrés Ibáñez, Francisco Melo, Antonio Galdámez Silva

**Affiliations:** 1 Departamento de Química, Facultad de Ciencias, Universidad de Chile, Las Palmeras 3425, Santiago 7800003, Chile; rvalenzuelafer@uchile.cl (R.A.V.-F.); alvceali@uchile.cl (Á.A.C.); 2 SMAT-C, Departamento de Física, Universidad de Santiago de Chile, Av. Víctor Jara 3493, Estación Central, Santiago 9160000, Chile; arianne.maine@usach.cl (A.M.); francisco.melo@usach.cl (F.M.); 3 Ciencias Básicas, Universidad Católica del Maule, Avenida San Miguel 3605, Talca 3480112, Chile; pbaraho@ucm.cl; 4 Institut des Sciences Chimiques de Rennes, UMR 6226, Université de Rennes, F-35042 Rennes, CEDEX, France; octavio.pena@hotmail.fr; 5 Departamento de Química Inorgánica, Facultad de Ciencias Químicas, Universidad Complutense, 28040 Madrid, Spain; ias@quim.ucm.es; 6 Departamento de Física, Facultad de Ciencias Físicas y Matemáticas, Universidad de Chile, Santiago 8330015, Chile; aibanez@dfi.uchile.cl

**Keywords:** spinels, inorganic materials, superparamagnetic behavior, lanthanides, ferrites

## Abstract

A new series of [Fe_3−_*_x_*Ln*_x_*]O_4_ nanoparticles, with Ln = Gd; Dy; Lu and *x* = 0.05; 0.1; 0.15, was synthesized using the coprecipitation method. Analyses by X-ray diffraction (XRD), Rietveld refinement, and high-resolution transmission electron microscopy (HRTEM) indicate that all phases crystallized in space group $Fd\bar{3}m$, characteristic of spinels. The XRD patterns, HRTEM, scanning electron microscopy analysis (SEM-EDS), and Raman spectra showed single phases. Transmission electron microscopy (TEM), Rietveld analysis, and Scherrer’s calculations confirm that these materials are nanoparticles with sizes in the range of ~6 nm to ~13 nm. Magnetic measurements reveal that the saturation magnetization (Ms) of the as-prepared ferrites increases with lanthanide chemical substitution (*x*), while the coercivity (Hc) has low values. The Raman analysis confirms that the compounds are ferrites and the Ms behavior can be explained by the relationship between the areas of the signals. The magnetic measurements indicate superparamagnetic behavior. The blocking temperatures (T_B_) were estimated from ZFC-FC measurements, and the use of the Néel equation enabled the magnetic anisotropy to be estimated.

## 1. Introduction

Understanding the magnetic behavior of a material is essential for discovering potential innovative applications. Among these, magnetic refrigerants stand out, harnessing the magnetocaloric effect to generate efficient cooling cycles [1]. Similarly, the hyperthermic effect has led to medical applications, such as oncological treatments through controlled temperature increases in nanoparticles [2]. In both cases, the key lies in the structural characterization and distribution of the elements that constitute the studied compounds. A prominent example of this approach includes magnetite (Fe_3_O_4_) and ferrites, a group of mixed iron oxides which are described by the chemical formula [Fe_3−_*_x_*M*_x_*]O_4_, where M can be a divalent cation transition metal [3]. These materials exhibit an inverse spinel-type structure and belong to the $Fd\bar{3}m$ space group [4].

Ferrite spinels, which are usually derivatives of ferrimagnetic ceramic compounds of substituted magnetite ${Fe}_{tet}^{3+}({Fe}^{2+}{Fe}^{3+}{)}_{Oct}O_{4}$, with cationic chemical substitutions of iron by cobalt, zinc, or manganese, have been studied. These metals have 2+ oxidation states. In the case of zinc, its configuration (d^10^) means it has a diamagnetic character; therefore, replacing Fe^2+^ (d^6^, paramagnetic) with Zn^2+^ influences the magnetic behavior of ferrite, since the random substitution of nonmagnetic ions produces the so-called Griffith effect, which leads to a decrease in the Curie temperature [5,6]. In the case of cobalt and manganese substitutions, these elements have a paramagnetic behavior (Co^2+^: d^7^; Mn^2+^: d^5^), so cobalt ferrites have a high coercivity while manganese substitution enhances magnetic saturation. Magnetic saturation increases as particle size rises [7]. Particle size has an important effect on the magnetic properties; for example, it can affect the magnitude of entropy [8], or superparamagnetic particles can be obtained [9].

The effect of substituting iron with other elements is of interest, and, in this case, the synthesis of ferrite nanoparticles is proposed, whereby iron is partially substituted by lanthanide cations to form [Fe_3−x_Ln_x_]O_4_, with Ln = Gd; Dy; Lu. Lanthanides were selected for their magnetic contribution. In the case of gadolinium [10], substitutions in iron oxides have been carried out, resulting in doped ferrites with hyperthermic characteristics [11]. On the other hand, dysprosium (Dy^3+^, *f*^9^) substitution could enhance the ferromagnetic character, as, based on the calculation of the magnetic moment, Dy^3+^ has the highest magnetic moment value among lanthanides (^6^H_15/2_, implying 10.63 MB). Lutetium (Lu^3+^, *f*^14^) has also been used because of its diamagnetic character, which could promote the Griffith effect.

In this study, from the chemical information on ferrites, rational synthesis of a family of compounds was performed to investigate the influence of the chemical substitution of lanthanides on their physical properties. The present work describes the synthesis and subsequent structural, microstructural, and spectroscopic characterizations, as well as the effect on the magnetic properties of ferrite nanoparticles where iron is partially substituted by lanthanide cations to form [Fe_3−_*_x_*Ln*_x_*]O_4_, with Ln = Gd; Dy; Lu. XRD, SEM-EDS, HRTEM-ED and Raman spectroscopy analyses were performed, and magnetic behavior was analyzed using hysteresis cycles and ZFC/FC curves.

## 2. Materials and Methods

### 2.1. Synthesis

Ferrite-type compounds were prepared by the coprecipitation method, for which the chlorides were used considering stoichiometry and oxidation states. Chloride salts of FeCl_2_·4H_2_O (≤100% purity, Sigma-Aldrich, Burlington, MA, USA), FeCl_3_·6H_2_O (≤100% purity, Sigma-Aldrich), GdCl_3_·6H_2_O (99% purity, Aldrich), DyCl_3_·6H_2_O (99% purity, Aldrich), and LuCl_3_·6H_2_O (99% purity, Aldrich) were mixed in stoichiometric proportions in 1 mol/L hydrochloric acid. After magnetic stirring at 70 °C for 1 h 30 min, during which 3 mol/L NaOH was added, a black‒brown precipitate was obtained. Vacuum filtration and washing with deionized water yielded the final products (Supplementary Information). To check the presence or absence of water, Fourier transform infrared (FTIR) analysis was performed, which did not show the characteristic signal of water, which should appear at ~3200 cm^−1^.

### 2.2. Characterization

Powder X-ray diffraction (PXRD) patterns were collected at room temperature on a Bruker D8 Advance diffractometer equipped with a Cu Kα radiation source (λ = 1.5406 Å) and scanned in the range 5° < 2θ < 80°. Rietveld refinement was carried out by TOPAS version 4.2 Bruker AXS software. The chemical compositions of the samples were determined by scanning electron microscopy using a Bruker Vega 3 Tescan system (SEM, JEOL 5400 system, Tokyo, Japan) equipped with a Quantax 400 microanalyzer energy-dispersive X-ray spectroscopy (EDS, Oxford LinK ISIS microanalyzer, Oxford Instruments, Abingdon, UK). Samples were mounted on double-sided carbon tape, which was adhered to an aluminum holder. The Raman measurements were undertaken with a confocal Raman Witec Alpha 300 microscope. An Ar laser with a 532 nm excitation wavelength, a 20× microscope objective with a numerical aperture of 0.75, and of the nanoparticles was recorded by a Hitachi HT7700 TEM (transmission and electrically cooled CCD camera were used for all samples at 1.3 mW. The spectral resolution was 4 cm^−1^, and 1000 scans per second were performed. The spectra were recorded from 100–1000 cm^−1^), enabling the visualization of structures with dimensions ranging from 0.2 to 100 nm. High-resolution transmission electron microscopy (HRTEM) and electron diffraction (ED) patterns were obtained using a JEOL JEM 3000 operating at an accelerating voltage of 300 kV. Samples were prepared by crushing the powders under n-butanol and dispersing them over copper grids covered with a porous carbon film. Semiquantitative chemical analyses were carried out using EDS.

Magnetic measurements were performed on pelletized powder samples using a Quantum Design, San Diego, CA, USA. The magnetic nature of the material was determined by zero-field-cooled/field-cooled (ZFC/FC) cycles at low fields (typically 50 Oe). Complementary magnetic measurements were carried out using a Quantum Design Dynacool Physical Property Measurement System (PPMS) for which the dc data were collected under an externally applied field of 100 Oe in the 1.8–300 K temperature range. Isothermal magnetization measurements were performed between −50 kOe and +50 kOe at 300 K.

## 3. Results and Discussion

### 3.1. Powder X-ray Diffraction (PXRD) and Electron Microscopy Characterization (SEM‒EDS and TEM)

**Figure 1:**
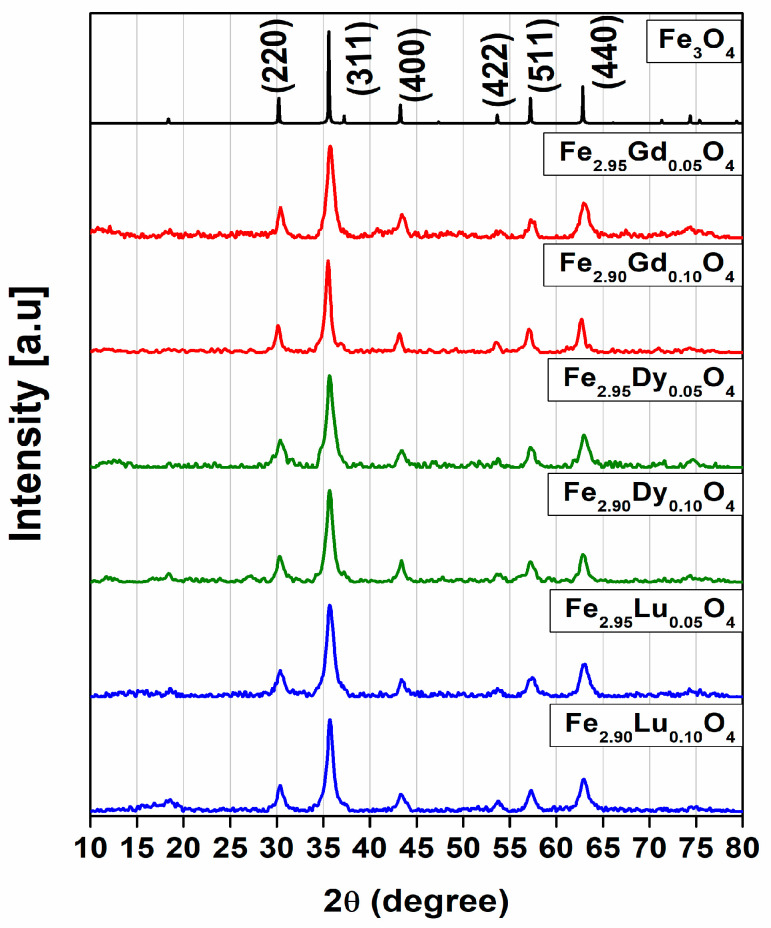
Powder XRD patterns at room temperature of substituted ferrites: [Fe_3−_*_x_*Ln*_x_*]O_4_ with Ln = Gd (red line); Dy(green line); and Lu (blue line). Fe_3_O_4_ magnetite end-member showing the corresponding *hkl* Miller indices (black line).

PXRD patterns and SEM‒EDS analyses indicate that the reaction products of the nominal composition of [Fe_3−_*_x_*Ln*_x_*]O_4_ Ln = Gd; Dy; Lu, *x* = 0.05; 0.1; 0.15 nanoparticles are single phases. The diffraction peaks can be indexed to the $Fd\bar{3}m$ space group characteristic of inverse spinel-type compounds [12]. At first, we performed chemical reactions for [Fe_3−x_Ln_x_]O_4_, with *x* = 0.05; 0.10; 0.15; 0.30; 0.40; and 0.50 compositions. When *x* > 0.15, chemical reaction products included >5% impurities. Figure 1 shows the powder patterns obtained for the magnetite and substituted compounds (*x* = 0.05 and 0.1) synthesized by the coprecipitation method. Rietveld refinement corroborates that all phases crystallized in space group $Fd\bar{3}m$, characteristic of inverse spinels, and provides information that is consistent with the XRD and Sherrer’s formula (see Supporting Information, Table S1 and Figure S1). Owing to the X-ray fluorescence and the low crystallinity of the samples, the cation distribution in the crystal structure cannot be discussed from the Rietveld refinement results.

Table 1 shows the lattice parameters and nanoparticle dimensions, as calculated by the Scherrer method. Despite the difference in the sizes of the cations—gadolinium (0.938 Å), dysprosium (0.912 Å), and lutetium (0.861 Å)—the *a*-cubic lattice parameters of ferrites decrease by ~1% in all cases, within the detection limits of the X-ray diffraction technique, compared to the nonsubstituted magnetite. The cell parameters do not obey Vegard´s law for any of the chemical compositions of the substitutions.

**Figure 2:**
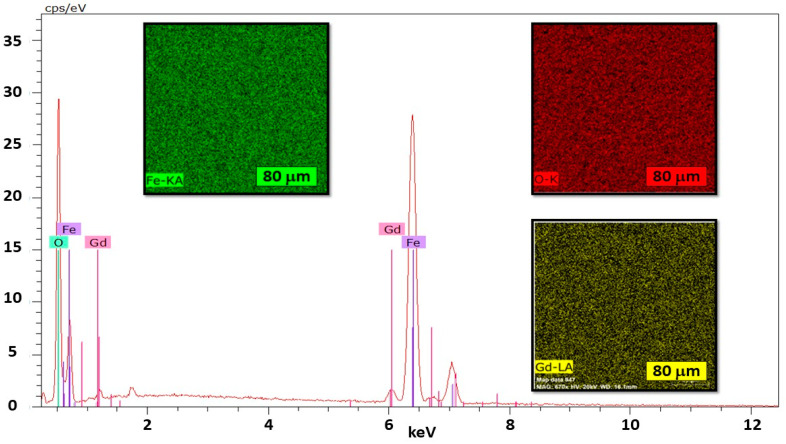
Representative SEM-EDS mapping images and X-ray spectra of powder sample of Fe_2.90_Gd_0.10_O_4_ (20 kV, 670×).

The backscattered image and EDS analysis reveal that the samples with nominal compositions of [Fe_3−_*_x_*Ln*_x_*]O_4_ Ln = Gd; Dy; Lu. *x* = 0.05; 0.1; 0.15 are uniform throughout the scanned region. The analysis of the distribution of the elements using the EDS-mapping technique confirm the homogeneity of the samples (Figure 2). The percentage differences between the theoretical chemical formula and those obtained from the EDS analyses are of the order of ~5%. In addition, HRTEM semiquantitative EDS spectra also indicate the same atomic percentages within experimental errors (see below). Similar results were obtained for all samples. Figures S2–S4 show representative EDS chemical mapping of [Fe_3−_*_x_*Dy*_x_*]O_4_ and [Fe_3−_*_x_*Lu*_x_*]O_4_ ferrites. Tables S2–S7 (Supplementary Information) show the chemical formula in relation to percentages in masses for ferrites.

### 3.2. Raman Spectra, TEM, and HRTEM Results

**Figure 3:**
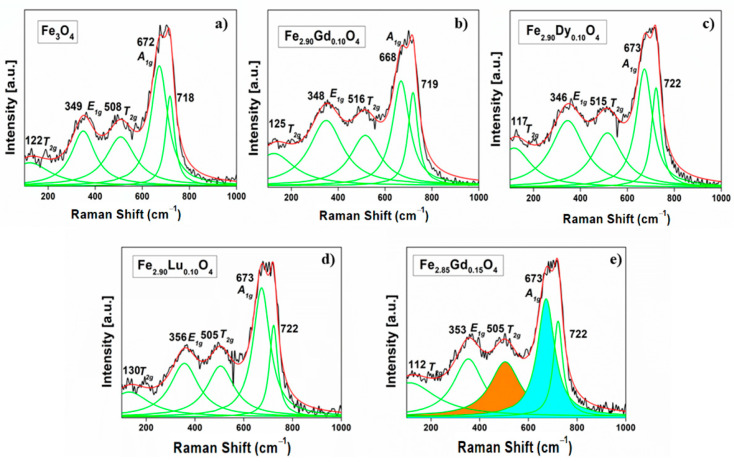
Raman spectra of ferrites with different contributions as deduced from fitting of peaks with Lorentzian curves (green lines): (**a**) Fe_3_O_4_, (**b**) Fe_2.90_Gd_0.10_O_4_, (**c**) Fe_2.90_Dy_0.10_O_4_, (**d**) Fe_2.90_Lu_0.10_O_4_, and (**e**) Fe_2.85_Gd_0.15_O_4_ with the area considered for the A_1g_/T_2g_ ratio (cyan and brown areas).

The Raman peaks were analyzed by fitting the spectra and subsequently identifying the vibrational modes by comparison with experimental and theoretical data for magnetite [13,14,15,16]. In our compounds, if a higher power of the laser had been applied to the particle, chemical transformation to another compound could have occurred (Figure S5). Indeed, Shebanova et al. [15] assigned this behavior to oxidation typical of a phase transition from ferrite to hematite. In our case, the optimized experimental conditions to measure the Raman spectra and avoid oxidation of the synthesized ferrites were a 532 nm laser with a power of 1.3 mW, acquiring one image per second with an accumulation of 1000 images (Figure S6). Figure 3 shows the fitting of the Raman spectra with Lorentzian curves for [Fe_3−_*_x_*Ln*_x_*]O_4_ samples between 100 and 1000 cm^−1^. The Raman spectra show three characteristic peaks, which can be assigned to the A_1g_ mode, where vibration can be viewed as a symmetric stretching of oxygen along the Fe-O bond. The E_1g_ and T_2g_ can be viewed as symmetric and asymmetric oxygen bonds, respectively, and the other two T_2g_ signals represent asymmetric stretching (Table 2). The other vibrational modes represent the translational movement of all Fe_3_O_4_ polyhedrons [14,17,18]. In the case of a $Fd\bar{3}m$ space group, an inversion center is present because of the centrosymmetric group, which implies the mutual exclusion of the Raman and infrared activities for the same vibrational modes. It is worth specifying which Raman peaks are associated with the different polyhedrons; the modes corresponding to octahedrons are present in the range of 460–660 cm^−1^, while the modes corresponding to tetrahedrons are those between 660 and 720 cm^−1^ [19].

In Figure 3a, the spectrum for the magnetite Fe_3_O_4_ shows two signals, at 122 and 718 cm^−1^. The first one is also assigned by several authors [17,19,20] as one of the *T_2g_* mode, while the one that appears at 718 cm^−1^ is assigned as part of a structural disorder [21]. The ratio of A_1g_/T_2g_ intensities, where the A_1g_ signal (~670 cm^−1^, belonging to the tetrahedral site) and the T_2g_ signal (~510 cm^−1^, belonging to the octahedral site) [21], for gadolinium-substituted Fe_2.95_Gd_0.05_O_4_, increases with respect to the proportions found in magnetite. This increase in intensity could be attributed to the preferential substitution of gadolinium in the structure in the tetrahedral site (Figure 3e). For the Fe_2.90_Gd_0.10_O_4_ phase, this ratio decreases, which could be due to the preferential distribution of Gd^3+^ in the octahedral site.

On the other hand, the signals of the tetrahedral site, shown as a second A_1g_ signal, could be associated with the presence of a second cation in this site. Nakagomi et al. synthesized MgFe_2_O_4_ ferrite, finding one band at ~720 cm^−1^, which was associated with A_1g_ due to the presence of a second type of cation. Indeed, Mg was preferentially located in the tetrahedral site and the signals were associated with the presence of both Fe-O and Mg-O bonds [22]. In a previous study, Sena et al. [10] identified ferrite substituted with gadolinium, where the gadolinium was in both octahedral and tetrahedral sites, as shown by Mossbauer techniques.

**Figure 4:**
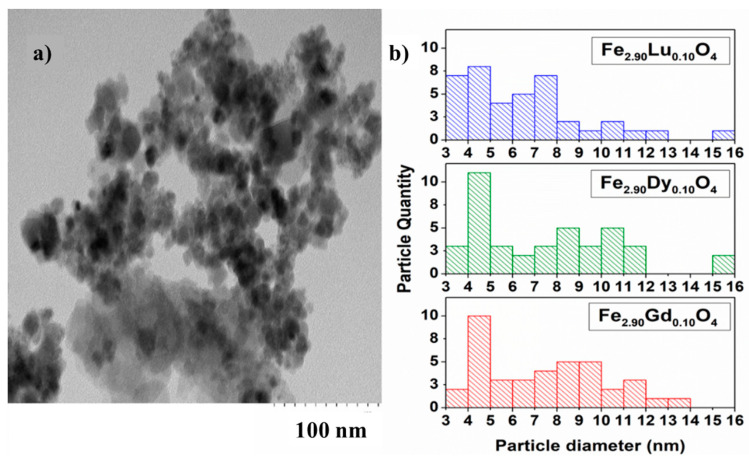
TEM and particle size distribution analysis. (**a**) Representative TEM image of Fe_2.90_Gd_0.10_O_4_ ferrite and (**b**) comparative histograms for ferrite (Fe_2.90_Ln_0.10_O_4_) particle sizes.

Figure 4 shows TEM images of Fe_2.90_Gd_0.10_O_4_ and histograms of the particle size distribution for all three Fe_2.90_Ln_0.10_O_4_ nanoparticles. Most were spherical in shape. Using histograms of 40 particles, the average particle diameter was 7.69 nm with a coefficient of variation of 0.404 nm. For Fe_2.90_Dy_0.10_O_4,_ the particle sizes correspond to an average of 7.55 nm with a coefficient of variation of 0.43 nm. Regarding ferrite with the formula Fe_2.90_Lu_0.10_O_4_, an average diameter of 6.421 nm was obtained with a coefficient of variation of 0.437 nm.

**Figure 5:**
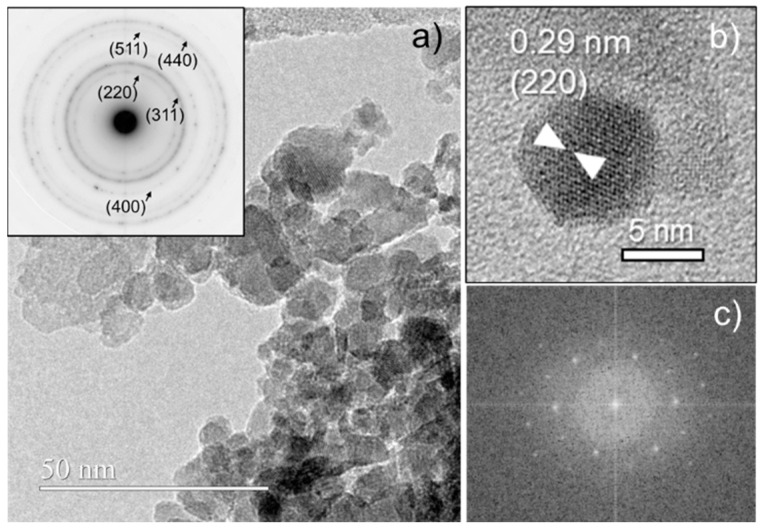
(**a**) Representative high-magnification image and corresponding ED pattern (inset), (**b**) detailed view of a particle showing (220) planes, and (**c**) corresponding FFT pattern, for Fe_2.95_Lu_0.05_O_4_.

The particle sizes are compared in Figure 4b, which presents the histograms of the particle size distribution obtained by TEM, as determined from the micrographs. Particles are generally smaller than 10 nm in diameter, while the ferrite with lutetium presents a tendency to be smaller, where its highest percentage is less than 4 nm. Additionally, the Scherrer calculation was performed from the XRD analyses to obtain the particle sizes for all compounds (Table 1); diameters differed from the TEM analysis by ~3 nm. Therefore, a further HRTEM analysis was carried out for the materials with *x* = 0.05, aiming to confirm the crystalline nature of the nanoparticles, their spinel-type structure, and their actual composition. Figure 5 shows some representative data for Fe_2.95_Lu_0.05_O_4_. As can be appreciated (Figure 5a), nanoparticles of about 8 nm appear, forming aggregate formations. The corresponding ED pattern is coherent with nanoparticles of spinel-type structure (inset in Figure 5a).

Figure 5b shows in detail one representative particle of 8.2 nm diameter, in which contrasts coherent with (220) planes of spinel-type structure are apparent. Its crystal nature is further confirmed by the corresponding fast Fourier transform (FFT, Figure 5c). Semiquantitative EDS spectra both of a and b regions indicate the same atomic percentage for Lu, suggesting good composition homogeneity. Therefore, these data enable us to confirm the crystal spinel structure and the composition of the Fe_2.95_Lu_0.05_O_4_ material. Nanoparticles of about 6–9 nm appear, forming aggregated formations in all Fe_2.95_Ln_0.05_O_4_ materials. Figure S7 shows representative low-magnification images and corresponding EDS spectra. Atomic percentages of 1.2–2.2% for Ln are obtained in all cases; hence, they are consistent with the nominal compositions within experimental error. Similar results were obtained for Fe_2.95_Dy_0.05_O_4_ and Fe_2.95_Lu_0.05_O_4_ materials, as observed in Figure S8.

### 3.3. Magnetic Properties

**Figure 6:**
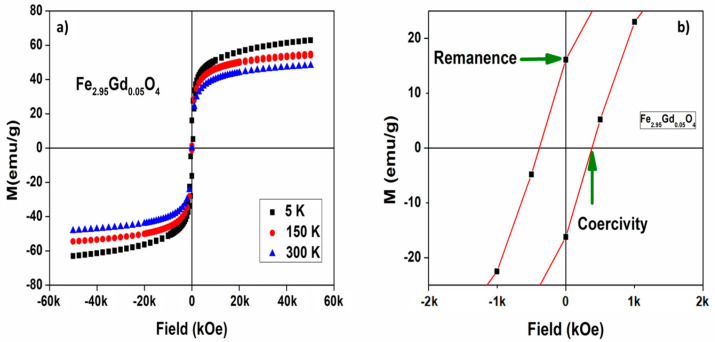
(**a**) Magnetic hysteresis plot at different temperatures for Fe_2.95_Gd_0.05_O_4_. (**b**) Remanence and coercivity determination at 5 K.

The magnetic characterization of [Fe_3−_*_x_*Ln*_x_*]O_4_ Ln = Gd; Dy; Lu. *x* = 0.05; 0.1; 0.15 was recorded using SQUID and PPMS equipment at 5 K, 150 K, and 300 K, with a maximum applied field up to ±50,000 Oe. ZFC/FC cycles were also recorded up to 400 K under low fields of 50 Oe. *M*–*H* curves for Fe_2.95_Gd_0.05_O_4_ are shown in Figure 6. The saturation magnetization (*Ms*), coercivity (*Ce*), and remanence (*Mr*) values calculated from the *M*–*H* curves are given in Table 3.

The magnetization curves in some samples exhibit approximately zero remanence and zero coercivity, which demonstrates that they are single-domain particles with superparamagnetic properties. The plots show the superparamagnetic nature of NPs at 300 K with negligible *Mr* values, consistent with a previous report where ferrite NPs also exhibited superparamagnetic behavior [23]. Fe_2.90_Gd_0.10_O_4_ and Fe_2.85_Lu_0.15_O_4_ show a soft ferrimagnetic nature, bordering on superparamagnetic-like. In the case of magnetite, this has the lowest values of remanence and coercivity; some phases that present substitutions have higher values for these two factors and for magnetic saturation.

[Fe_3−_*_x_*Ln*_x_*]O_4_ with Ln = Gd and Lu ferrites show the highest saturation, with values ranging from 43 to 65 emu/g and 43 to 56 emu/g, respectively. Surprisingly, [Fe_3−_*_x_*Dy*_x_*]O_4_, from which we expected highest magnetic saturation due to the dysprosium magnetic moment, has the lowest values (38–56 emu/g). The experimental values of the samples reported in Table 3 did not show a direct relationship with the molar amount of substituted lanthanides. Nanocrystals of magnetite (Fe_3_O_4_) prepared by alkaline precipitation have saturation values of 51.68 emu/g at 300 K [24], while in another report using a N_2_ atmosphere during synthesis, 67.3 emu/g was obtained [25]. Previous research on magnetite materials obtained saturation values of 62, 70, and 73 emu/g, with the variation being attributed to particle size [26]. Nanoclusters show values of 65 emu/g for magnetite in the form of nanoparticles, while the “bulk magnetites” present values of 92 emu/g [27]. Solvent-free synthesis of ~9 nm nanoparticles had a magnetic saturation of 76 emu/g [28], while Guardia et al. obtained a value of 82 emu/g for “bulk magnetite” [29]. In contrast, several experimental studies on [Fe_3-*x*_M*_x_*]O_4_, with M = transition metals, have suggested that the saturation values depend on the chemical substitution in the crystal structure. For example, nanocrystals of nonstoichiometric cobalt ferrite reported by Ngo et al. [30] show values of 44 and 56 emu/g, which are directly related to particle size. Sharifi et al. [3] informed saturation values of 56 to 80 emu/g for CoFe_2_O_4_, NiFe_2_O_4_, and MnFe_2_O_4_. Therefore, the magnetic saturation values for ferrites [Fe_3-*x*_Ln*_x_*]O_4_ reported in this work (Table 3) are all lower than those of [Fe_3−_*_x_*M*_x_*]O_4_ with M = transition metals.

**Figure 7:**
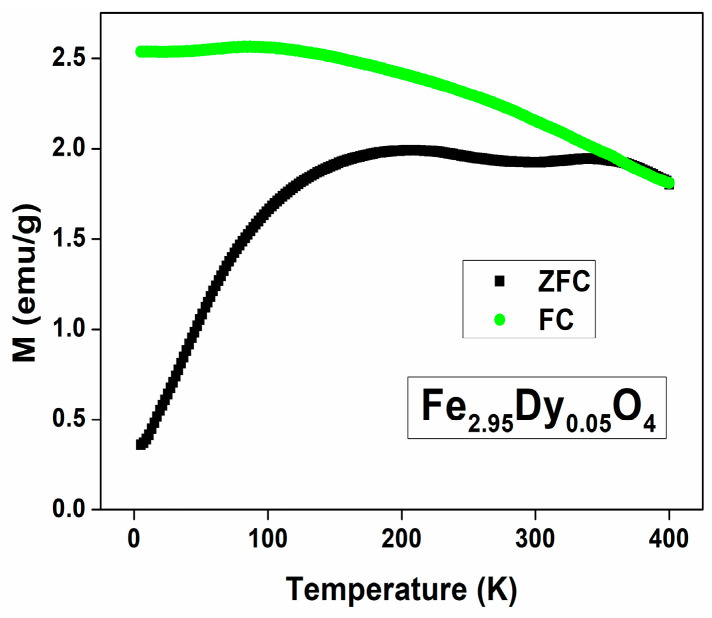
Zero-field-cooled (ZFC) and field-cooled (FC) against temperature plots of Fe_2.95_Dy_0.05_O_4_ at a magnetic field H = 50 Oe.

**Figure 8:**
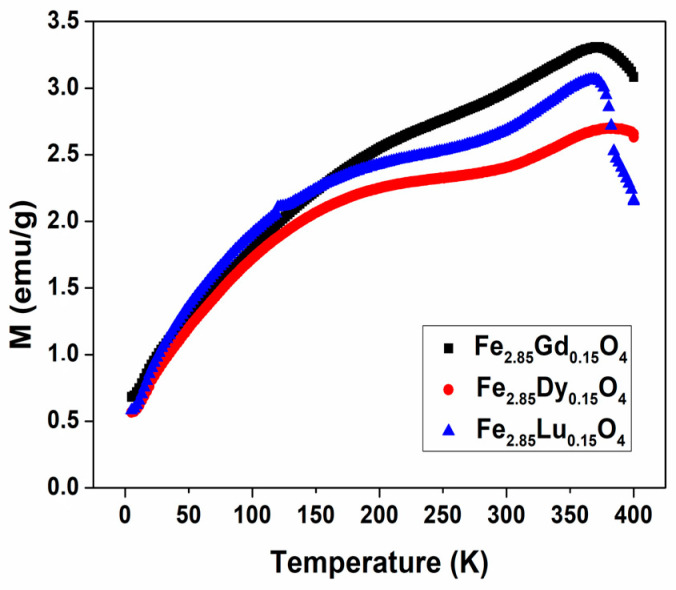
Zero-field-cooled (ZFC) and field-cooled (FC) against temperature plots of Fe_2.85_M_0.15_O_4_ (M = Gd; Dy; and Lu) at H = 50 Oe.

Figure 7 shows representative curves of the magnetic susceptibility variation as a function of temperature in the range 5 to 400 K under an external magnetic field of 50 Oe, as recorded in zero-field-cooled (ZFC) and field-cooled (FC) conditions. From the curves, the superimposition of the ZFC and FC curves at temperatures above 330 K is clearly observed. Figure 8 shows the ZFC curves for gadolinium, dysprosium, and lutetium ferrites with 0.15 substitution. To calculate the magnetic anisotropy, it is necessary to determine the blocking temperature, which was obtained by means of the cooling curves by identifying the first peak which presents a descent, as seen in Figure 8. In the case of ferrite with gadolinium substitutions, the blocking temperature occurs at 354.39 K at 50 Oe, so its volume, assuming a spherical shape, is 1.41 × 10^−24^ m^3^; from this figure, we can determine the value of the magnetic anisotropy ordered using the Néel relaxing time equation. An anisotropy value of 8.54 × 10^4^ J/m^3^ for Fe_2.90_Gd_0.10_O_4_ was obtained, which is the lowest value of all compounds synthesized here, while the other ferrites have values around 10^5^ J/m^3^, with the highest ones being for lutetium ferrites.

In the case of the magnetites, anisotropy values of 1.1 × 10^4^ J/m^3^ at 280 K were found, and, in general, for this compound, reported values are within the range of 10^4^ J/m^3^, as in the aforementioned article by Guardia et al. [29]. Maldonado-Camargo et al. obtained figures between 20 and 70 KJ/m^3^ [31], while Suto et al. observed values of 30 KJ/m^3^ [2]. For nanoparticles with different shapes, Mamiya presented values between 10 and 20 KJ/m^3^ [32]. However, it is also possible to find values of the order of 10^5^ J/m^3^, as in the case of Barnakov et al. [33], Řezníček et al. [34], and Lisjak et al. [35]. Table 4 shows the values obtained for the compounds generated in this study.

The theory of single domains, known as the remanence ratio, according to Stoner-Wohlfarth, relates remanence and magnetic saturation (M_r_/M_s_) [36,37]. For a ratio value of 0.5, anisotropy presents a uniaxial character, while for a value of 0.832, it is cubic. Table 3 shows the results obtained in our study, all of which are below 0.5. Therefore, such uniaxial anisotropy represents contributions of the spins at the surface and the nucleus of the particles, which are not necessarily equal [38].

This effect is also related to magnetization since it originates from the contribution of both the nucleus and the surface of the nanoparticle, known as core–shell magnetization. The behavior of the surface spins is different from those found in the nucleus of the nanoparticle, since the superficial spins are disordered reducing magnetization, whilst the critical temperature of the magnetic order of the nanoparticles differentiates the magnetic behavior of a nanoparticle from the bulk material. Experimentally, the blocking temperatures of the compounds generated in this study are all above 300 K (Table 4).

The A_1g_/T_2g_ ratio of the Raman spectra correlates with magnetic saturation; in the case of the gadolinium ferrites, when this ratio increases, so does magnetic saturation (Table 4). Lutetium ferrites present a similar behavior, that is, when the A_1g_/T_2g_ ratio rises, the magnetic saturation and anisotropy both increase. Unexpectedly, lutetium ferrites present high values of magnetic saturation M_s_ in comparison to the other ferrites; this behavior can be attributed to its diamagnetic character, since in this case, the only ions providing a magnetic contribution are the iron ions themselves. In other words, the magnetic moment is not counteracted by lutetium since there are no elements with an antiparallel magnetic moment in the tetrahedral and octahedral sites.

## 4. Conclusions

[Fe_3−_*_x_*Ln*_x_*]O_4_ Ln = Gd; Dy; Lu with *x* = 0.05; 0.1; 0.15 were synthesized by a coprecipitation method. They crystallize in spinel-type structures, as determined by PXRD, Raman, and HRTEM analysis. The Raman spectra show signals which are assigned to *E_1g_*, *3T_2g_*, and *A_1g_* vibrational modes. The backscattered images and the EDS (SEM and HRTEM) elemental analyses demonstrate that the elements in the powder samples are uniformly distributed. The TEM analyses carried out on some of the samples indicate that the size of the samples is nanometric and that they present a small difference in size with respect to the calculation made by the Scherrer equation.

The magnetic susceptibility measurements demonstrate that the nanoparticles possess superparamagnetic behaviors, with an increase in magnetic saturation in the case of gadolinium and lutetium with respect to those of the pristine compound, while the values of saturation are comparable for dysprosium. The behavior of ferrites with dysprosium can be explained by the presence of the lanthanide in the octahedral and tetrahedral sites, with spins located antiparallel to the iron ions, canceling the magnetic contribution. The distribution of the elements in both sites was estimated from Raman spectra by means of the A_1g_/T_2g_ ratio of the characteristic signals associated with the tetrahedral and octahedral sites. This ratio is related to magnetic saturation and magnetic remanence, and shows uniaxial anisotropy for all ferrites synthesized. The analysis of magnetic susceptibility under a field of 50 Oe allowed us to determine the blocking temperatures, from which it was possible to calculate the anisotropy of the ferrites, giving values of common magnitude for this type of compounds.

## Figures and Tables

**Table 1:** Unit cell parameters and crystallite size of [Fe_3−_*_x_*Ln*_x_*]O_4_ and pristine magnetite.

Chemical Formula	*a* (Å)	Volume (Å^3^)	Diameter (nm)
Fe_3_O_4_ *	8.39	591.96	-
Fe_3_O_4_^ζ^	8.33 (1)	580.8 (2)	10.9 (3)
Fe_2.95_Gd_0.05_O_4_	8.35 (2)	583.6 (6)	10.5 (1)
Fe_2.90_Gd_0.10_O_4_	8.37 (1)	588.3 (3)	11.2 (8)
Fe_2.85_Gd_0.15_O_4_	8.37 (1)	586.8 (2)	12.5 (3)
Fe_2.95_Dy_0.05_O_4_	8.38 (2)	584.8 (1)	8.8 (8)
Fe_2.90_Dy_0.10_O_4_	8.38 (3)	588.7 (5)	10.7 (1)
Fe_2.85_Dy_0.15_O_4_	8.38 (3)	589.4 (1)	11.5 (1)
Fe_2.95_Lu_0.05_O_4_	8.37 (3)	589.3 (7)	9.71 (2)
Fe_2.90_Lu_0.10_O_4_	8.39 (1)	587.3 (3)	9.98 (1)
Fe_2.85_Lu_0.15_O_4_	8.39 (2)	587.2 (2)	12.3 (3)

* Reference [12]. ^ζ^ This work.

**Table 2:** Comparison of vibrational modes for ferrite with lanthanide substitutions.

		Vibrational Mode (cm^−1^)			
Phase	T_2g_	E_1g_	T_2g_ (2)	A_1g_	A_1g_ (2)
Fe_3_O_4_	122	349	508	672	718
Fe_2.95_Gd_0.05_O_4_	122	355	510	675	721
Fe_2.90_Gd_0.10_O_4_	125	348	516	668	719
Fe_2.85_Gd_0.15_O_4_	112	353	505	673	722
Fe_2.95_Dy_0.05_O_4_	127	348	510	671	719
Fe_2.90_Dy_0.10_O_4_	117	346	515	673	722
Fe_2.85_Dy_0.15_O_4_	n/d	347	515	673	716
Fe_2.95_Lu_0.05_O_4_	125	354	506	669	721
Fe_2.90_Lu_0.10_O_4_	130	356	505	673	722
Fe_2.85_Lu_0.15_O_4_	129	349	506	672	722

**Table 3:** Magnetic saturation, magnetic remanence, and coercivity of all ferrites at 5 K, 150 K, and 300 K.

Replaced Element	Phase	Temperature	Saturation (emu/g)	Remanence (emu/g)	Coercivity (Oe)	Mr/Ms
	Fe_3_O_4_	5	48.90	15.25	370	0.312
		150	45.45	0.53	11.55	0.012
		300	38.62	0.0016	0.0064	0.0004
Gadolinium	Fe_2.95_Gd_0.05_O_4_	5	62.97	16.05	379	0.255
		150	54.12	1.4	47.96	0.026
		300	48.20	0.055	2.27	0.001
	Fe_2.90_Gd_0.10_O_4_	5	63.57	16.38	339.16	0.258
		150	47.92	3.38	111.76	0.071
		300	43.39	1.31	47.08	0.030
	Fe_2.85_Gd_0.15_O_4_	5	65.83	17.53	386.08	0.266
		150	52.79	3.67	111.52	0.070
		300	45.99	0.71	24.76	0.015
Dysprosium	Fe_2.95_Dy_0.05_O_4_	5	49.92	13.36	407.41	0.268
		150	44.08	1.11	51.44	0.025
		300	38.24	0.26	14.78	0.007
	Fe_2.90_Dy_0.10_O_4_	5	52.89	14.22	457.53	0.269
		150	45.51	2.21	60.27	0.049
		300	38.34	0.43	21.18	0.011
	Fe_2.85_Dy_0.15_O_4_	5	56.61	15.78	434.67	0.279
		150	44.21	2.86	73.57	0.065
		300	38.60	0.75	33.59	0.0194
Lutetium	Fe_2.95_Lu_0.05_O_4_	5	51.84	17.05	389.09	0.329
		150	48.66	1.02	26.26	0.021
		300	46.91	0.18	7.75	0.004
	Fe_2.90_Lu_0.10_O_4_	5	56.59	16.9	355.7	0.299
		150	56.01	2.27	49.11	0.041
		300	49.43	0.42	14.94	0.009
	Fe_2.85_Lu_0.15_O_4_	5	50.42	17.46	367.24	0.346
		150	49.43	3.34	71.65	0.068
		300	43.32	0.95	35.89	0.022

**Table 4:** Relation between magnetic properties and ratio of the Raman peak areas, blocking temperatures, and anisotropies.

Phases	Ms (emu/g)	*A_1g_*/*T_2g_*	*A_1g_*/*E_1g_*	Diameter (nm)	T_B_ (K)	Anisotropy (J/m^3^)
Fe_3_O_4_	38.62	1.40	1.54	10.9 (3)	300	1.45 × 10^5^
Fe_2.95_Gd_0.05_O_4_	48.20	1.60	1.64	10.5 (1)	364.11	2.02 × 10^5^
Fe_2.90_Gd_0.10_O_4_	43.39	1.19	0.82	11.2 (8)	354.39	8.44 × 10^4^
Fe_2.85_Gd_0.15_O_4_	45.99	1.23	1.18	12.5 (3)	369.62	1.22 × 10^5^
Fe_2.95_Dy_0.05_O_4_	38.24	1.61	1.45	8.8 (8)	340.74	3.26 × 10^5^
Fe_2.90_Dy_0.10_O_4_	38.34	1.17	0.87	10.7 (1)	358.32	1.90 × 10^5^
Fe_2.85_Dy_0.15_O_4_	38.60	1.43	0.84	11.5 (1)	382.98	1.61 × 10^5^
Fe_2.95_Lu_0.05_O_4_	46.91	1.51	1.29	9.71 (2)	327.89	2.31 × 10^5^
Fe_2.90_Lu_0.10_O_4_	49.43	1.57	1.44	9.98 (1)	372.6	2.46 × 10^5^
Fe_2.85_Lu_0.15_O_4_	43.32	1.24	1.17	12.3 (3)	368.3	1.30 × 10^5^

## Data Availability

Data are contained within the article.

## Supplementary Materials

The following supporting information can be downloaded at: https://www.mdpi.com/article/10.3390/nano14110971/s1, Figure S1: Observed, calculated, and difference XRD profiles of Fe_2.95_Gd_0.05_O_4_ and Fe_2.95_Lu_0.05_O_4_ fitted using the Rietveld method with TOPAS software; Figure S2: Mapping analysis representing a homogeneous distribution of the elements of (a) Fe_2.95_Gd_0.05_O_4_ and (b) Fe_2.85_Gd_0.15_O_4_ ferrites; Figure S3: Mapping analysis representing a homogeneous distribution of the elements of (a) Fe_2.95_Dy_0.05_O_4_, (b) Fe_2.90_Dy_0.10_O_4_, and (c) Fe_2.85_Dy_0.15_O_4_ ferrites; Figure S4: Mapping analysis representing a homogeneous distribution of the elements of (a) Fe_2.95_Lu_0.05_O_4_, (b) Fe_2.90_Lu_0.10_O_4_, and (c) Fe_2.85_Lu_0.15_O_4_ ferrites; Figure S5: Raman spectrum of compounds half-transformed from ferrite to hematite; Figure S6: Raman spectra of ferrites with lanthanide substitutions; Figure S7: Representative low-magnification images and corresponding EDS spectra for Fe_2.95_Ln_0.05_O_4_; Figure S8: Representative high-magnification images and corresponding ED patterns for (a) Fe_2.95_Dy_0.05_O_4_ and (b) Fe_2.95_Gd_0.05_O_4_; Figure S9: Magnetic hysteresis graph at 300 K for Fe_2.90_Lu_0.10_O_4_ ferrite with different diameters; Table S1: *R*-indices, space group, crystallinity, and size obtained from Rietveld refinement of PXRD patterns using TOPAS-Bruker software; Table S2: Chemical formula in relation to percentages of the masses for ferrite with gadolinium substitutions; Table S3: Comparison of element percentages for ferrite with gadolinium substitutions; Table S4: Chemical formula in relation to percentages of the masses for ferrite with dysprosium substitutions; Table S5: Comparison of element percentages for ferrite with dysprosium substitutions; Table S6: Chemical formula in relation to percentages of the masses for ferrite with lutetium substitutions; Table S7: Comparison of element percentages for ferrite with lutetium substitutions; Table S8: Magnetic saturation, magnetic remanence, and coercivity of different-sized ferrites with formula Fe_2.90_M_0.10_O_4_ with M = Gd, Dy, and Lu; Table S9: Ratio of the Raman peak areas of the ferrite signals; Figure S10: Steps in ferrite synthesis.
