# Supplementary material for: Structural Characterization and Magnetic Behavior Due to the Cationic Substitution of Lanthanides on Ferrite Nanoparticles
Source: Nanomaterials (Basel). 2024 Jun 3;14(11):971. doi: 10.3390/nano14110971 (PMC11173717; doi:10.3390/nano14110971)
Supplement: Supplementary file 1 [file nanomaterials-14-00971-s001.zip › nanomaterials-2981618-supplementary.pdf]

Supplementary Information

Structural Characterization and Magnetic Behavior Due to the Cationic Substitution of Lanthanides on Ferrite Nanoparticles

Cristóbal Pinto García [1,*], Arianne Maine [2], Rodrigo A. Valenzuela-Fernández [1], Álvaro Aliaga Cerón [1], Patricia Barahona Huenchumil [3], Octavio Peña [4], Inmaculada Álvarez-Serrano [5], Andrés Ibáñez [6], Francisco Melo [2] and Antonio Galdámez Silva [1,*]

Rietveld results

Table S1. *R*-indices, space group, crystallinity and size obtained from Rietveld refinement of PXRD patterns using TOPAS-Bruker software.

Phase name	R-Bragg	Space group	Crystallite size (nm)	Degree of crystallinity (%)
$Fe_{2.95}Gd_{0.05}O_4$	0.177	$Fd\bar{3}m$	12.2	11
$Fe_{2.95}Lu_{0.05}O_4$	0.133		11.4	24

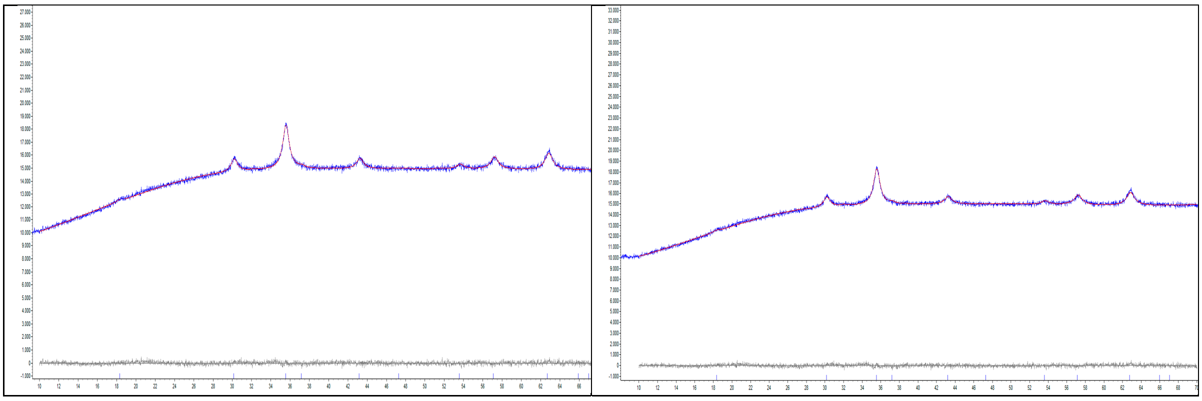

Figure S1. Observed, calculated, and difference XRD profiles of $Fe_{2.95}Gd_{0.05}O_4$ and $Fe_{2.95}Lu_{0.05}O_4$ fitted using the Rietveld method with TOPAS software.

SEM Analysis

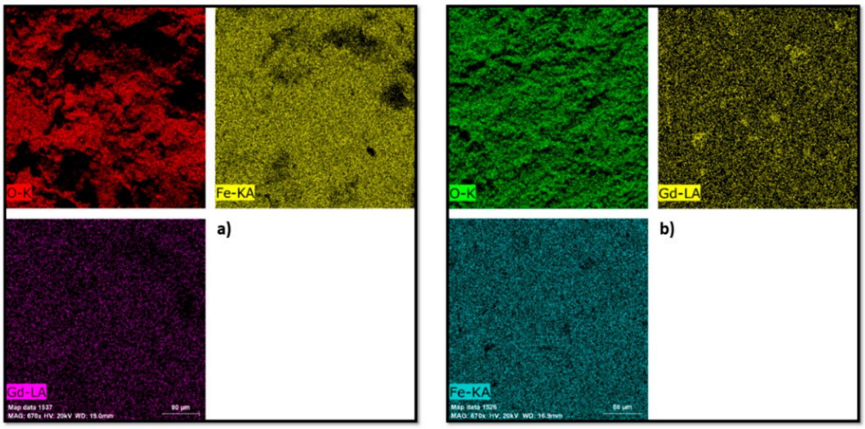

Figure S2: Mapping analysis representing a homogeneous distribution of the elements of a) $Fe_{2.95}Gd_{0.05}O_4$ and b) $Fe_{2.85}Gd_{0.15}O_4$ ferrites.

Table S2: Chemical formula in relation to percentages of the masses for ferrite with gadolinium substitutions

Phase	Analysis	Fe	Gd	O
**$Fe_{2.95}Gd_{0.05}O_4$**	General	2.95	0.06	3.18
	Punctual 1	2.95	0.06	2.60
	Punctual 2	2.95	0.05	3.31
	Punctual 3	2.95	0.06	2.56
**$Fe_{2.90}Gd_{0.10}O_4$**	General	2.90	0.09	4.76
	Punctual 1	2.90	0.10	4.74
	Punctual 2	2.90	0.09	4.80
	Punctual 3	2.90	0.09	4.74
**$Fe_{2.85}Gd_{0.15}O_4$**	General	2.85	0.15	3.75
	Punctual 1	2.85	0.15	3.78
	Punctual 2	2.85	0.14	3.78
	Punctual 3	2.85	0.14	3.80

Table S3: Comparison of element percentages for ferrite with gadolinium substitutions.

	Element	Chemistry Formula percentage	General	Punctual 1	Punctual 2	Punctual 3
**$Fe_{2.95}Gd_{0.05}O_4$**	Fe	6.63	73.05	76.45	72.88	76.64
	Gd	3.32	4.15	4.28	3.67	4.31

	O	27.05	22.58	19.27	23.45	19.05
**$Fe_{2.90}Gd_{0.10}O_4$**	Fe	67.01	64.02	63.96	64.10	64.18
	Gd	6.51	5.85	6.07	5.54	5.78
	O	26.48	30.13	29.96	30.37	30.04
**$Fe_{2.85}Gd_{0.15}O_4$**	Fe	67.01	66.22	66.05	65.91	65.90
	Gd	6.51	8.81	8.85	9.05	8.95
	O	26.48	24.97	25.10	25.04	25.15

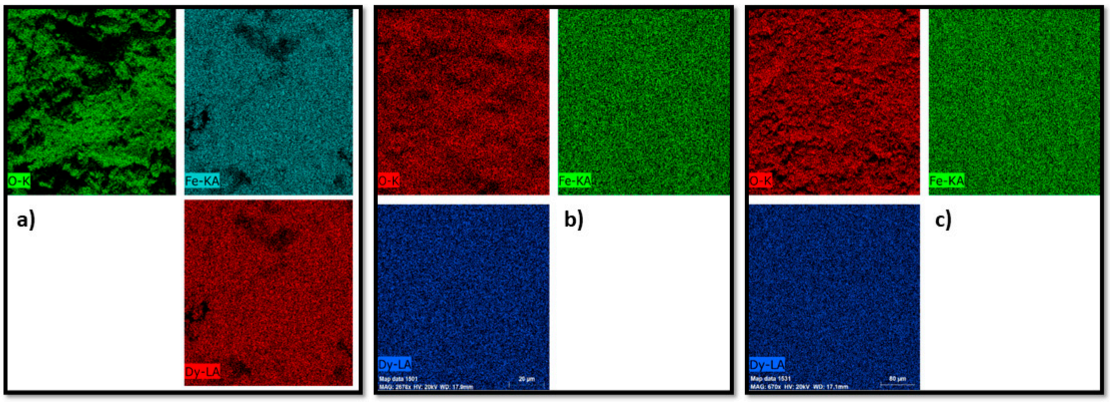

Figure S3: Mapping analysis representing a homogeneous distribution of the elements of a) $Fe_{2.95}Dy_{0.05}O_4$, b) $Fe_{2.90}Dy_{0.10}O_4$ and c) $Fe_{2.85}Dy_{0.15}O_4$ ferrites.

Table S4: Chemical formula in relation to percentages of the masses for ferrite with dysprosium substitutions

Phase	Analysis	Fe	Gd	O
**$Fe_{2.95}Gd_{0.05}O_4$**	General	2.95	0.05	3.31
	Punctual 1	2.95	0.06	2.97
	Punctual 2	2.95	0.06	4.23
	Punctual 3	2.95	0.06	4.22
**$Fe_{2.90}Gd_{0.10}O_4$**	General	2.90	0.11	4.53
	Punctual 1	2.90	0.11	4.36
	Punctual 2	2.90	0.12	4.76
	Punctual 3	2.90	0.11	4.73
**$Fe_{2.85}Gd_{0.15}O_4$**	General	2.85	0.12	4.38
	Punctual 1	2.85	0.13	4.32
	Punctual 2	2.85	0.12	4.18
	Punctual 3	2.85	0.13	4.47

Table S5: Comparison of element percentages for ferrite with dysprosium substitutions.

	Element	Chemistry formula percentage	General	Punctual 1	Punctual 2	Punctual 3
**$Fe_{2.95}Dy_{0.05}O_4$**	Fe	69.55	72.42	72.93	66.57	66.29
	Dy	3.43	3.53	4.26	3.74	4.18
	O	27.02	23.30	21.02	27.33	27.16
**$Fe_{2.90}Dy_{0.10}O_4$**	Fe	66.87	64.00	65.01	62.71	63.50
	Dy	6.71	7.35	6.98	7.80	6.86
	O	26.42	28.65	28.02	29.64	29.64
**$Fe_{2.85}Dy_{0.15}O_4$**	Fe	64.30	63.87	63.70	64.71	63.28
	Dy	9.85	8.00	8.66	8.12	8.29
	O	25.85	28.13	27.64	27.17	28.43

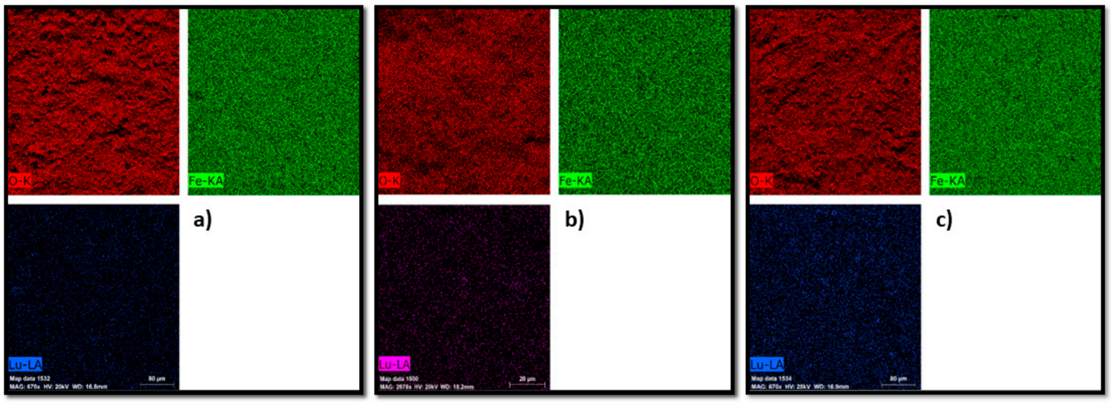

Figure S4: Mapping analysis representing a homogeneous distribution of the elements of a) $Fe_{2.95}Lu_{0.05}O_4$, b) $Fe_{2.90}Lu_{0.10}O_4$ and c) $Fe_{2.85}Lu_{0.15}O_4$ ferrites.

Table S6: Chemical formula in relation to percentages of the masses for ferrite with lutetium substitutions

Phases	Analysis	Fe	Lu	O
**$Fe_{2.95}Lu_{0.05}O_4$**	General	2.95	0.04	4.03
	Punctual 1	2.95	0.04	3.89
	Punctual 2	2.95	0.04	4.04
	Punctual 3	2.95	0.04	3.96
**$Fe_{2.90}Lu_{0.10}O_4$**	General	2.90	0.10	4.22

	Punctual 1	2.90	0.10	4.04
	Punctual 2	2.90	0.10	4.47
	Punctual 3	2.90	0.10	3.93
**$Fe_{2.85}Lu_{0.15}O_4$**	General	2.85	0.11	4.46
	Punctual 1	2.85	0.12	4.26
	Punctual 2	2.85	0.12	4.51
	Punctual 3	2.85	0.24	4.57

Table S7: Comparison of element percentages for ferrite with lutetium substitutions.

	Element	Chemistry formula percentage	General	Punctual 1	Punctual 2	Punctual 3
**$Fe_{2.95}Lu_{0.05}O_4$**	Fe	69.37	69.66	70.31	69.46	69.74
	Lu	3.68	3.09	2.92	2.99	3.09
	O	26.95	27.25	26.53	27.25	26.85
**$Fe_{2.90}Lu_{0.10}O_4$**	Fe	66.53	65.39	66.22	64.46	66.78
	Lu	7.19	7.34	7.34	7.06	7.27
	O	26.29	27.26	26.43	28.49	25.95
**$Fe_{2.85}Lu_{0.15}O_4$**	Fe	63.82	65.03	64.17	65.22	64.88
	Lu	10.52	8.93	9.25	8.91	9.30
	O	25.66	26.03	26.58	25.88	25.82

Raman Analysis

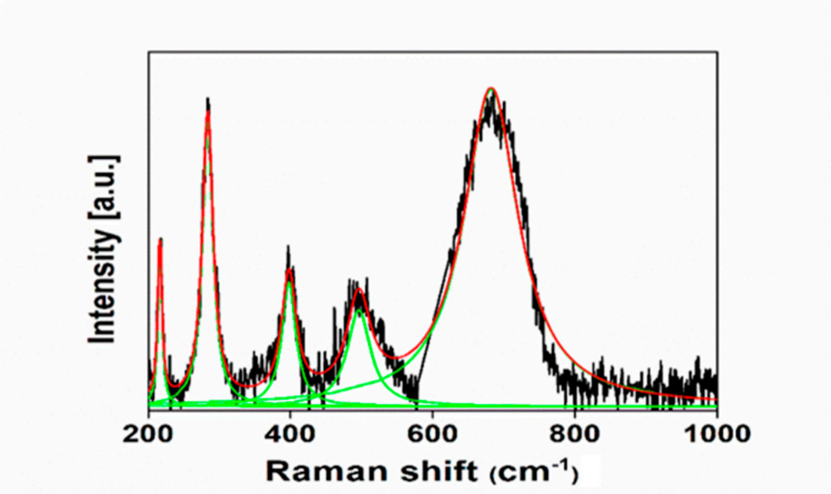

Figure S5: Raman spectrum of compounds half-transformed from ferrite to hematite.

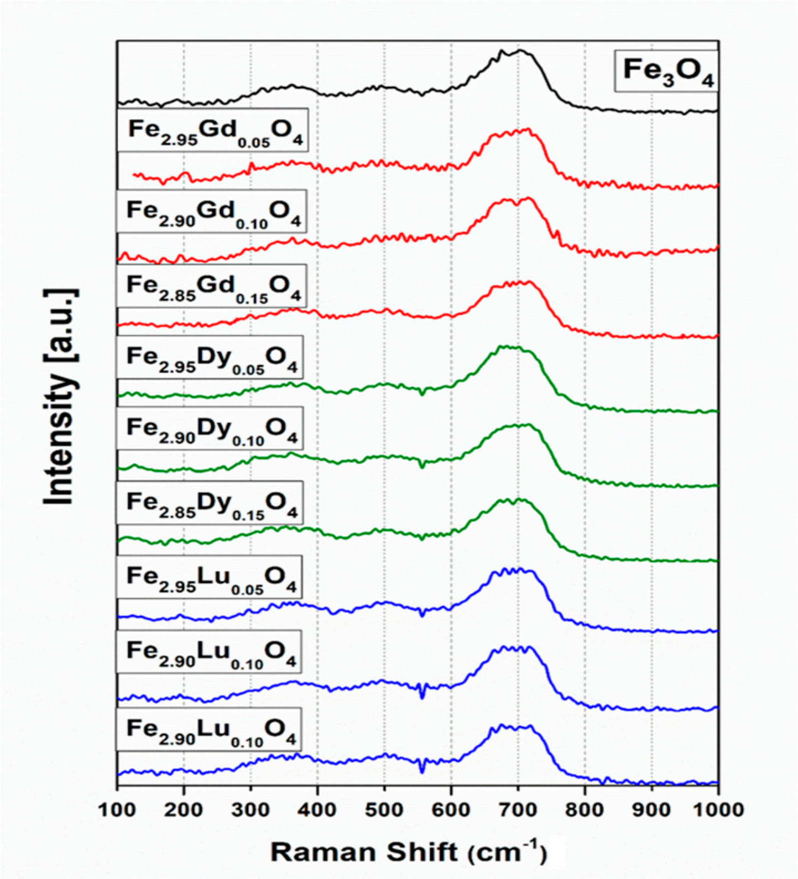

Figure S6: Raman spectra of ferrites with lanthanide substitutions.

HRTEM, ED patterns and EDS spectra analysis

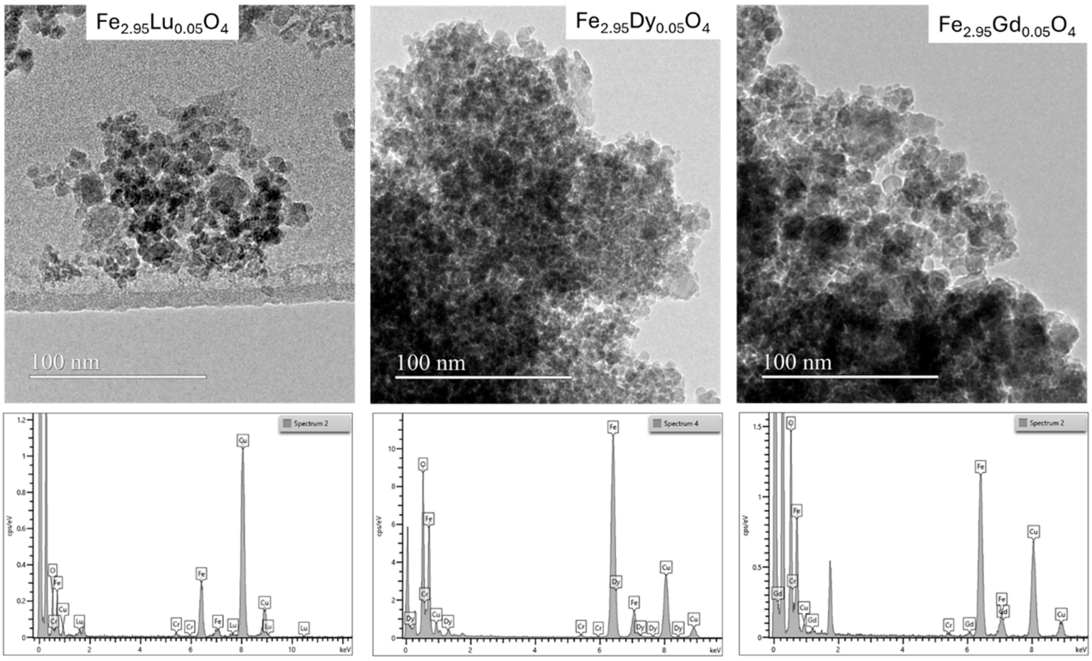

Figure S7. Representative low magnification images and corresponding EDS spectra for $Fe_{2.95}Ln_{0.05}O_4$

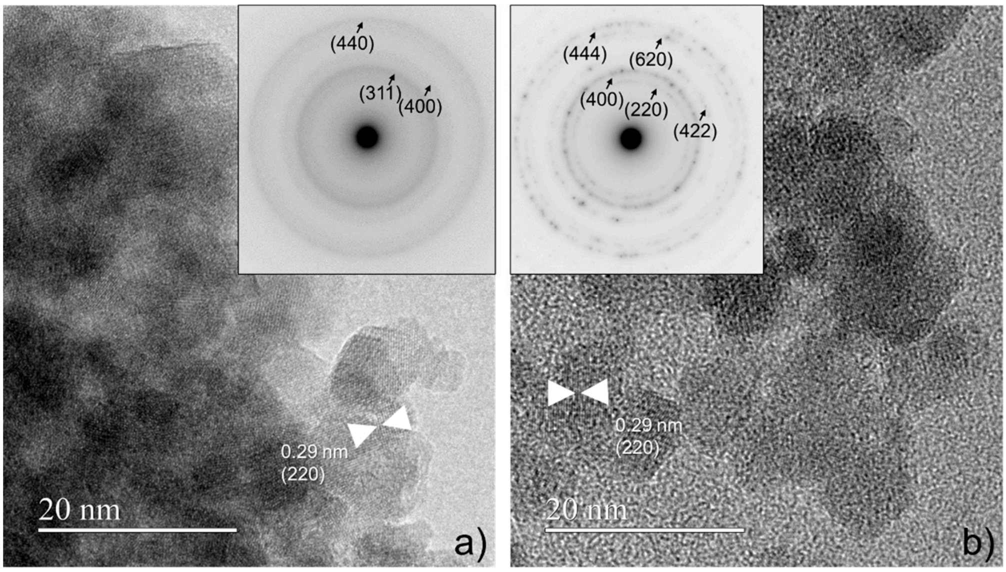

Figure S8. Representative high magnification images and corresponding ED patterns for a) $Fe_{2.95}Dy_{0.05}O_4$ and b) $Fe_{2.95}Gd_{0.05}O_4$

Magnetic behavior

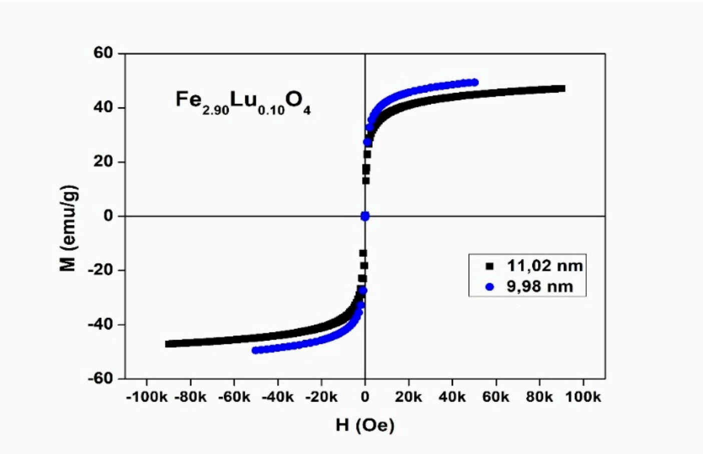

Figure S9: Magnetic hysteresis graph at 300 K for $Fe_{2.90}Lu_{0.10}O_4$ ferrite with different diameters.

Table S8: Magnetic saturation, magnetic remanence and coercivity of different-sized ferrites with formula $Fe_{2.90}M_{0.10}O_4$ with M = Gd, Dy and Lu

Phase	Diameter (nm)	Saturation (emu/g)	Remanence (emu/g)	Coercivity (Oe)
$Fe_{2.90}Gd_{0.10}O_4$	11.24	52.01	4.24	73.50
	13.92	43.39	1.31	47.08
$Fe_{2.90}Dy_{0.10}O_4$	10.08	45.93	1.25	30.60
	10.69	38.34	0.43	21.18
$Fe_{2.90}Lu_{0.10}O_4$	11.02	47.25	2.76	123.5
	9.98	49.43	0.42	14.94

Table S9: Ratio of the Raman peak areas of the ferrite signals.

	A_{1g}/T_{2g}	$A_{1g}(2)/A_{1g}$	E_{1g}/T_{2g}	A_{1g}/E_{1g}
Fe_3O_4	1.60	0.57	0.98	1.64
$Fe_{2.95}Gd_{0.05}O_4$	1.19	0.51	1.44	0.82
$Fe_{2.90}Gd_{0.10}O_4$	1.23	0.44	1.04	1.18
$Fe_{2.85}Gd_{0.15}O_4$	1.61	0.38	1.11	1.45
$Fe_{2.95}Dy_{0.05}O_4$	1.17	0.48	1.36	0.87
$Fe_{2.90}Dy_{0.10}O_4$	1.43	0.47	1.70	0.84
$Fe_{2.85}Dy_{0.15}O_4$	1.51	0.49	1.17	1.29
$Fe_{2.95}Lu_{0.05}O_4$	1.57	0.34	1.09	1.44
$Fe_{2.90}Lu_{0.10}O_4$	1.24	0.42	1.06	1.17
$Fe_{2.85}Lu_{0.15}O_4$	1.60	0.57	0.98	1.64

Experimental details

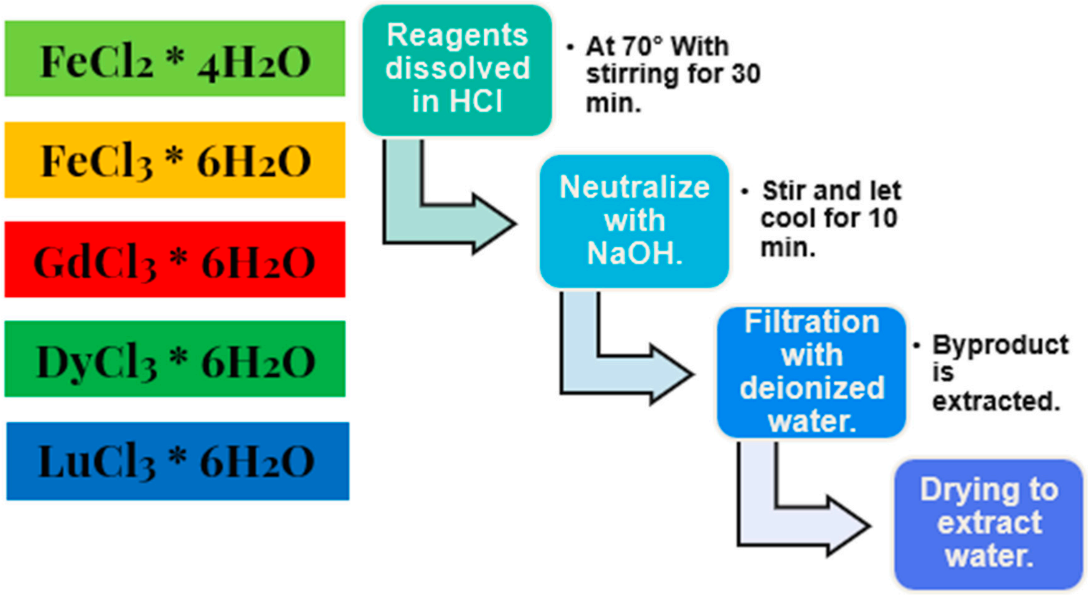

Figure S10: Steps in ferrite synthesis.
